# Ceftolozane/Tazobactam Resistance and Mechanisms in Carbapenem-Nonsusceptible Pseudomonas aeruginosa

**DOI:** 10.1128/mSphere.01026-20

**Published:** 2021-01-27

**Authors:** Jocelyn Qi-Min Teo, Jie Chong Lim, Cheng Yee Tang, Shannon Jing-Yi Lee, Si Hui Tan, James Heng-Chiak Sim, Rick Twee-Hee Ong, Andrea Lay-Hoon Kwa

**Affiliations:** a Department of Pharmacy, Singapore General Hospital, Singapore, Singapore; b Saw Swee Hock School of Public Health, National University of Singapore and National University Health System, Singapore, Singapore; c Department of Pharmacy, National University of Singapore, Singapore, Singapore; d Department of Microbiology, Singapore General Hospital, Singapore, Singapore; e Singhealth Duke-NUS Medicine Academic Clinical Programme, Singapore, Singapore; f Emerging Infectious Diseases, Duke-National University of Singapore Medical School, Singapore, Singapore; Antimicrobial Development Specialists, LLC

**Keywords:** *Pseudomonas aeruginosa*, ceftolozane/tazobactam, molecular characterization

## Abstract

This study established the *in vitro* activity of ceftolozane/tazobactam (C/T) and its genotypic resistance mechanisms by whole-genome sequencing (WGS) in 195 carbapenem-nonsusceptible Pseudomonas aeruginosa (CNSPA) clinical isolates recovered from Singapore between 2009 and 2020. C/T susceptibility rates were low, at 37.9%. Cross-resistance to ceftazidime/avibactam was observed, although susceptibility to the agent was slightly higher, at 41.0%. Whole-genome sequencing revealed that C/T resistance was largely mediated by the presence of horizontally acquired *β*-lactamases, especially metallo-*β*-lactamases. These were primarily disseminated in well-recognized high-risk clones belonging to sequence types (ST) 235, 308, and 179. C/T resistance was also observed in several non-carbapenemase-producing isolates, in which resistance was likely mediated by *β*-lactamases and, to a smaller extent, mutations in AmpC-related genes. There was no obvious mechanism of resistance observed in five isolates. The high C/T resistance highlights the limited utility of the agent as an empirical agent in our setting. Knowledge of local molecular epidemiology is crucial in determining the potential of therapy with novel agents.

**IMPORTANCE** Pseudomonas aeruginosa infection is one of the most difficult health care-associated infections to treat due to the ability of the organism to acquire a multitude of resistance mechanisms and express the multidrug resistance phenotype. Ceftolozane/tazobactam (C/T), a novel *β*-lactam/*β*-lactamase inhibitor combination, addresses an unmet medical need in patients with these multidrug-resistant P. aeruginosa infections. Our findings demonstrate geographical variation in C/T susceptibility owing to the distinct local molecular epidemiology. This study adds on to the growing knowledge of C/T resistance, particularly mutational resistance, and will aid in the design of future β-lactams and *β*-lactamase inhibitors. WGS proved to be a useful tool to understand the P. aeruginosa resistome and its contribution to emerging resistance in novel antimicrobial agents.

## Abstract (precis)

Pseudomonas aeruginosa infection is one of the most difficult health care-associated infections to treat due to the ability of the organism to acquire a multitude of resistance mechanisms and express the multidrug resistance phenotype. Ceftolozane/tazobactam (C/T), a novel *β*-lactam/*β*-lactamase inhibitor combination, addresses an unmet medical need in patients with these multidrug-resistant P. aeruginosa infections.

## INTRODUCTION

Pseudomonas aeruginosa is one of the most common pathogens implicated in hospital-acquired infections (1). Aside from its intrinsic resistance to several antibiotics, its propensity to acquire resistance is responsible for its multidrug resistance profile, rendering the pathogen a therapeutic challenge (2). Carbapenems are the drugs of choice in the management of severe P. aeruginosa infections. Unfortunately, resistance to this class of agents has developed, resulting in carbapenem-nonsusceptible P. aeruginosa (CNSPA). Carbapenem nonsusceptibility rates in clinical P. aeruginosa isolates at Singapore General Hospital have hovered at approximately 8 to 10% since 2011 (3). This is congruent to the nation’s overall carbapenem resistance rate in P. aeruginosa clinical isolates derived from public hospitals (https://www.moh.gov.sg/resources-statistics/reports/one-health-report-on-antimicrobial-utilisation-and-resistance-2017). Additionally, carbapenem nonsusceptibility was detected in 24% of P. aeruginosa hospital-acquired infections in Singapore (1).

Ceftolozane/tazobactam (C/T) is a novel broad-spectrum new-generation cephalosporin/*β*-lactamase inhibitor combination that is highly active against P. aeruginosa. This novel agent has been designed to “escape” many of P. aeruginosa’s common resistance mechanisms, including AmpC hydrolysis, drug efflux, and OprD porin inactivation (2, 4, 5). Several studies have also demonstrated high C/T susceptibilities against CNSPA, supporting its empirical use in such infections, in which most other antibiotics are rendered ineffective (6–8). However, antibiotic susceptibilities are subject to geographical and institutional variations. The lack of local surveillance data has limited our understanding of the clinical utility of C/T in the local context. The objectives of this study were to establish the *in vitro* activity of C/T in a collection of CNSPA isolates recovered from Singapore and to characterize the genotypic profiles of C/T-nonsusceptible CNSPA.

(This study was presented in part at the 29th European Congress of Clinical Microbiology & Infectious Diseases, Amsterdam, Netherlands, 13 to 16 April 2019 [P1334].)

## RESULTS AND DISCUSSION

### Antimicrobial susceptibility profiles.

A total of 195 CNSPA isolates were included in the study. Only 74 (37.9%) isolates were susceptible (inhibited at <8 mg/liter). Table 1 shows the susceptibility patterns for various antibiotics against CNSPA. C/T demonstrated better activity than the other *β*-lactams, with the exception of ceftazidime/avibactam (CZA), which had a slightly higher susceptibility rate (41.0%). Only 66 (33.8%) isolates were susceptible to both C/T and CZA. Considerably higher susceptibility rates were observed for the non β-lactam antibiotics, such as amikacin (58.0%). Resistance remained rare for polymyxin B (3.1%).

**TABLE 1:** Activities of antimicrobial agents against 195 clinical isolates of carbapenem-nonsusceptible Pseudomonas aeruginosa

Antimicrobial agent	MIC (mg/liter)			Susceptibility^*a*^		
	50%	90%	Range			
				% S	% I	% R
Ceftolozane/tazobactam^*b*^	≥128/4	≥128/4	≤0.5/4 to ≥128/4	37.9	3.1	59.0
Other β-lactam agents						
Imipenem	32	≥64	1 to ≥64	3.6	8.7	87.7
Meropenem	32	≥64	≤0.25 to ≥64	13.3	8.2	78.5
Doripenem	32	≥64	≤0.25 to ≥64	16.4	8.7	74.9
Aztreonam	32	≥128	4 to ≥128	13.9	22.0	64.1
Cefepime	≥128	≥128	≤1 to ≥128	17.4	10.8	71.8
Piperacillin/tazobactam	128/4	≥256/4	4 to ≥256/4	13.9	8.7	77.4
Ceftazidime/avibactam^*b*^	32/4	≥128/4	1/4 to ≥128/4	41.0		59.0
Other classes						
Amikacin	8	≥128	≤1 to ≥128	58.0	6.1	35.9
Gentamicin^*c*^				35.4	2.0	62.6
Levofloxacin	32	≥64	≤0.25 to ≥64	18.0	8.7	73.3
Polymyxin B	1	2	≤0.25 to ≥32		96.9	3.1

a S, susceptible; I, intermediate; R, resistant.

b Susceptibility was determined using gradient MIC test strips.

c Susceptibility was determined using disk diffusion or Vitek routinely at the microbiology laboratory.

Whole-genome sequencing (WGS) revealed carbapenemase production among 86 of the 195 isolates (44.1%); all 86 were nonsusceptible to C/T, as expected (C/T MIC_50_, ≥128/4 mg/liter; MIC_90_, ≥128/4 mg/liter). Hence, all 74 C/T-susceptible CNSPA isolates were observed in the 109 remaining non-carbapenemase-producing isolates, resulting in a susceptibility rate of 67.9% (C/T MIC_50_, 2/4 mg/liter; MIC_90_, ≥128/4 mg/liter) in this cohort.

The low C/T susceptibility rate (37.9%) is in contrast to several other studies conducted elsewhere, in which moderate to high susceptibility rates (ranging from 67 to 88%) were observed in multidrug-resistant or carbapenem-resistant P. aeruginosa (9–11). Even among non-carbapenemase-producing CNSPA isolates, moderate C/T susceptibility (67.9%) was observed. This suggests that C/T has limited utility as an empirical agent for suspected P. aeruginosa hospital-acquired infections in our setting, and susceptibility testing for the agent or knowledge of carbapenemase status is imperative prior to its use. Notably, most of the C/T-nonsusceptible isolates were recovered prior to the introduction of C/T into our institution, even among non-carbapenemase-producing CNSPA, substantiating that drivers of C/T resistance are likely not limited to C/T usage.

### Genomic profiles of 121 C/T-nonsusceptible P. aeruginosa isolates.

A brief summary of the genomic characteristics of all 195 CNSPA isolates is presented in Table 2. C/T nonsusceptibility was detected in 22 sequence types (STs) (21 known STs and 1 novel ST) in the 121 C/T-nonsusceptible CNSPA isolates. C/T resistance in the high-risk clones of ST235 (46/121 [38.0%]) and ST308 (33/121 [27.3%]) were the most prevalent. ST175 P. aeruginosa, the international high-risk clone with AmpC hyperproduction plus OprD inactivation which has been associated with C/T resistance, was not found in our study (12). In contrast, the ST types in C/T-susceptible isolates were even more widely distributed (58 different STs).

**TABLE 2:** Genotypic characteristics of 195 CNSPA isolates

Parameter	C/T-nonsusceptible isolates (*n* = 121)		C/T-susceptible isolates (*n* = 74)
	Carbapenemase producers (*n* = 86)	Non-carbapenemase producers (*n* = 35)	
No. of STs	12	14 (13 + 1 new)	58 (55 + 3 new)
Known STs^*a*^	**233**, 235, 244, **308**, 316, 357, **621**, 773, **823**, **964**, **3440**, **3444**	155, **179**, 235, 244, **252**, 274, **313**, **316**, 357, **664**, 815, 1076, **1666**	11, 17, 27, 111, 155, 207, 235, 244, 245, 253, 266, 274, 292, 298, 314, 357, 389, 408, 446, 463, 471, 485, 508, 553, 560, 564, 569, 606, 620, 645, 697, 708, 773, 792, 815, 840, 882, 1076, 1247, 1649, 1930, 2013, 2021, 2033, 2069, 2326, 2476, 2651, 3078, 3311, 3439, 3442, 3443, 3445, 3446
Harbors acquired *β*-lactamase	10	24	5
AmpC and regulator alteration^*b*^	10	6	
PBP3 alteration^*b*^	3	1	
Ceftazidime/avibactam susceptible	4	10	66

a STs in bold were observed only in the C/T-nonsusceptible population in our study.

b Only alterations unique to the C/T-nonsusceptible population are reported. Refer to Fig. 1 and 2 for the specific observed alterations for each isolate.

We analyzed the resistance mechanisms for the 121 C/T-nonsusceptible isolates. Figures 1 and 2 depict the isolates’ characteristics and the potential mechanisms responsible for C/T nonsusceptibility in 86 carbapenemase-producing and 35 non-carbapenemase-producing CNSPA isolates, respectively. C/T nonsusceptibility can be explained primarily by the presence of horizontally acquired carbapenemases in a large proportion of the C/T-nonsusceptible isolates (86/121 [71.1%]). The predominant types of genes detected were metallo-*β*-lactamases: *bla*_NDM_ (35 isolates), *bla*_IMP_ (31 isolates), and *bla*_VIM_ (11 isolates). Carbapenem-hydrolyzing *bla*_GES-5_ accounted for the remaining isolates, with the exception of two isolates which harbored *bla*_KPC-2_ and *bla*_OXA-232_.

**FIG 1:**
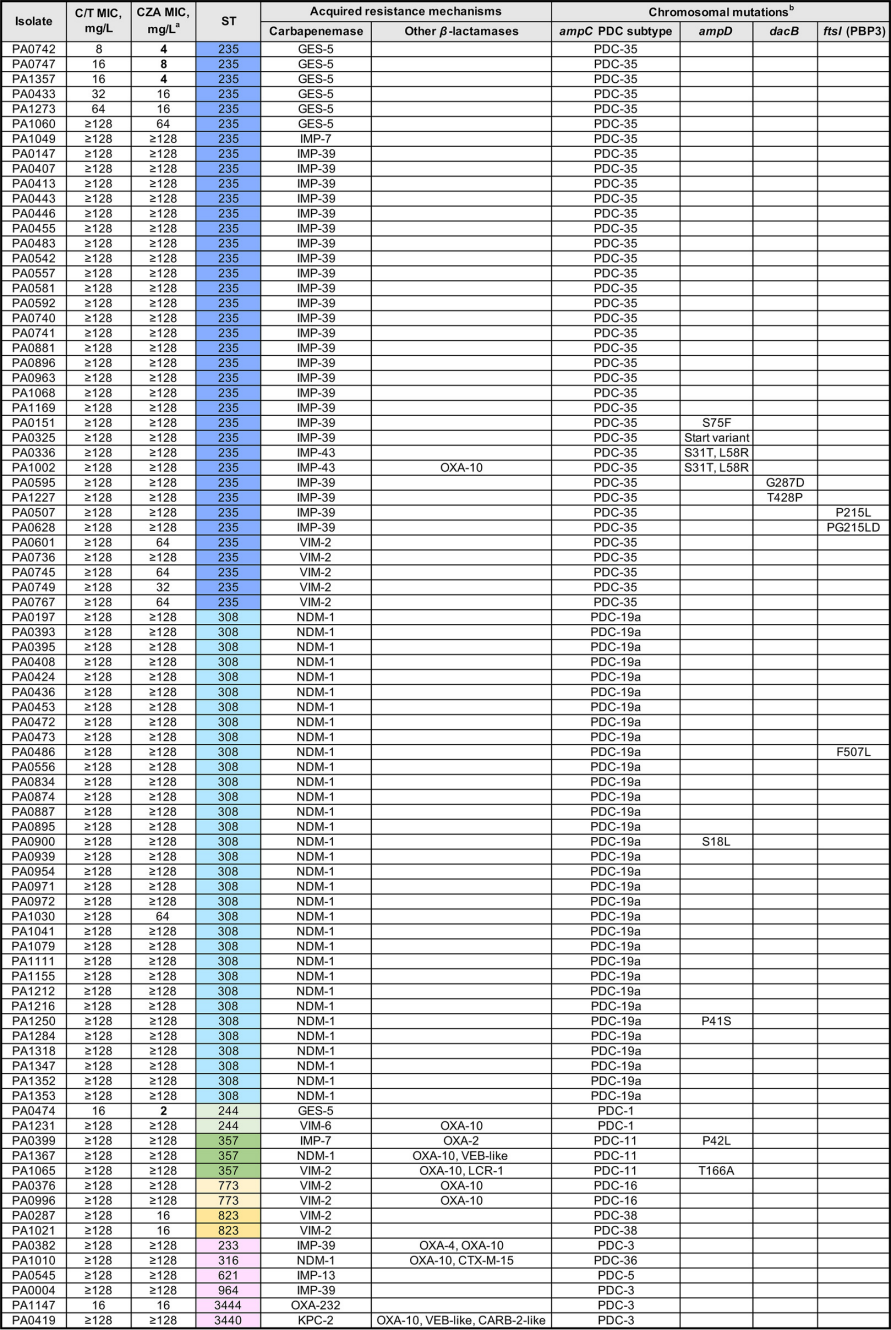
Mechanisms of ceftolozane/tazobactam (C/T) resistance in 86 carbapenemase-producing CNSPA isolates. a, Bold values indicate ceftazidime/avibactam (CZA) susceptibility. b, The main chromosomal mutations (*ampC*, *ampR*, *dacB*, and *ftsI*) leading to amino acid substitutions compared to the reference wild-type comparator amino acid sequences from Pseudomonas aeruginosa PAO1 are shown. The list of nonsynonymous variations were refined to include only those more likely to be involved in the C/T-resistant phenotype, i.e., (i) mutations with known effect on resistance according to published evidence and (ii) mutations with predicted functional impact (i.e., deleterious) and not identified in wild-type/susceptible isolates. There were no mutations found in *ampR* in this set of isolates. PDC, *Pseudomonas*-derived cephalosporinase; ST, sequence type.

**FIG 2:**
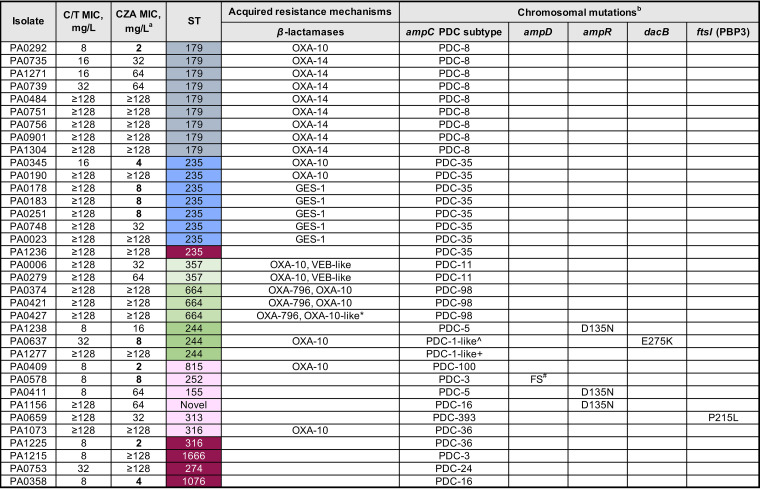
Mechanisms of C/T resistance in 35 non-carbapenemase-producing CNSPA isolates. a, Bold values indicate CZA susceptibility. b, The main chromosomal mutations (*ampC*, *ampR*, *dacB*, and *ftsI*) leading to amino acid substitutions compared to the reference wild-type comparator amino acid sequences from Pseudomonas aeruginosa PAO1 are shown. The list of nonsynonymous variations was refined to include only those more likely to be involved in the C/T-resistant phenotype, i.e., (i) mutations with known effect on resistance according to published evidence and (ii) mutations with predicted functional impact (i.e., deleterious) and not identified in wild-type/susceptible isolates. *, G439C amino acid substitution in OXA-10; ^^^, A163T amino acid substitution in AmpC; ^+^, ΔK74-E75 in AmpC; ^#^, frameshift (FS) at position 149.

In the 35 non-carbapenemase-producing C/T-nonsusceptible CNSPA isolates, horizontally acquired extended-spectrum *β*-lactamases (ESBLs) were frequently observed (24/35 [68.6%]). Notably, *bla*_OXA-14_, the extended-spectrum variant of *bla*_OXA-10_ which has been associated with C/T resistance (13), was detected in eight isolates, all of which were ST179. *bla*_VEB_, *bla*_GES-1_, *bla*_OXA-10_, and other *bla*_OXA_ variants were also detected. C/T appeared to have variable activity in P. aeruginosa with secondary ESBLs. Various ESBLs such as those encoded by *bla*_GES_ and *bla*_VEB_ have been shown to inactivate C/T (14).

We noted that the distribution of these exogenous *β*-lactam resistance elements was limited primarily to three main clones, ST235 (*n* = 46), ST308 (*n* = 33), and ST179 (*n* = 9), which accounted for 72.7% of the C/T-nonsusceptible isolates. Within each clone, there was little or no intraclonal variation. This suggests that multidrug resistance, including C/T resistance, is contributed primarily by a limited number of clones which have gained a strong foothold in our setting, although P. aeruginosa organisms of other diverse STs could also acquire these ESBLs/carbapenemases over time, resulting in broad-spectrum resistance.

As ceftolozane is neither affected by efflux pumps nor transported via OprD, resistance is driven primarily by acquisition of ESBLs, AmpC hyperproduction, AmpC structural modifications, or mutations in PBP3 (15, 16). Since we observed a number of C/T-nonsusceptible isolates without any carbapenemases/ESBLs or harboring only narrow-spectrum beta-lactamases like *bla*_OXA-10_, we analyzed the chromosomal genes related to AmpC and its expression (*ampC* and the regulator genes *ampD*, *ampR*, and *dacB*), as well as the *ftsI* gene (encoding PBP3), which is the target binding site of C/T. Our analysis revealed that most of the 121 C/T-nonsusceptible CNSPA isolates harbored single nucleotide polymorphisms (SNPs) resulting in nonsynonymous AmpC amino acid substitutions. The number of amino acid substitutions ranged from 0 to 5 (median, 5). This is congruent to the high sequence polymorphism of AmpC reported for P. aeruginosa (16). Classification of the isolates based on *Pseudomonas*-derived cephalosporinase (PDC) subtypes showed a total of 17 different subtypes. The PDC-35 subtype was the most prevalent; it was detected solely in the 46 ST235 isolates. This was followed by PDC-19a, which was found exclusively in the 33 ST308 isolates. The majority of these AmpC amino acid substitutions were unlikely to be associated with C/T nonsusceptibility, as they were either similarly found in the susceptible strains in our study or have been described for wild-type strains elsewhere. We did not detect any SNPs implicated in C/T nonsusceptibility which had been described in literature previously (4, 16, 17). However, we did observe potentially deleterious variants (A163T in PA0637 and a 2-amino-acid deletion, K74-E75, in PA1277) in two isolates (Fig. 2). Deleterious SNPs in the other *ampD*, *ampR*, and *dacB* regulator genes and PBP3 variants were infrequently observed, occurring in only approximately 16% of the isolates. There were five nonsusceptible isolates (highlighted in dark purple in Fig. 2) which did not appear to have any ESBLs/carbapenemases or alterations in AmpC or PBP3. The identified deleterious SNPs in this study have not been reported in the literature, and thus, their role in mediating C/T resistance requires further validation.

Although the aim of this study did not include a detailed investigation of the mechanisms of CZA resistance, we noted that there were differential susceptibilities in the two agents. Cross-resistance was high due to the high prevalence of metallo*-β*-lactamases, which both agents were inactive against. In contrast to tazobactam, avibactam was designed to have potent activity against class C *β*-lactamases and have a slightly broader anti *β*-lactamases activity (inclusive of KPC and OXA-48) (14, 17). This could explain the observation of the 14 (11.6%) isolates among the 121 C/T-nonsusceptible CNSPA isolates which remained susceptible to CZA (Table 2). These isolates primarily harbored GES and OXA *β*-lactamases (Fig. 1 and 2), which can be inhibited by avibactam. Additionally, 8 (10.8%) of the 74 C/T-susceptible CNSPA were resistant to CZA, which had moderate MICs near the breakpoint (16 mg/liter). We postulate that resistance in these isolates may be attributed to drug efflux and/or decreased cell permeability in the presence of low levels of AmpC overexpression which may still be overcome by C/T (18–20). Nevertheless, due to the slight difference in the activities of the two agents, there may be a role for each agent, depending on the molecular epidemiology of the setting.

### Concluding remarks.

CNSPA is a major treatment challenge due to a lack of available effective agents. Novel agents such as the AmpC-stable C/T are introduced in a bid to expand the armamentarium against these difficult-to-treat organisms. In this study, we assessed the rates of *in vitro* susceptibility to C/T and the molecular mechanisms mediating C/T resistance in CNSPA recovered from a large tertiary hospital in Singapore where C/T has only recently (January 2019) been introduced into its formulary.

The observed high C/T nonsusceptibility rates in our CNSPA, together with cross-resistance to CZA, the other novel *β*-lactamase inhibitor combination, signify a severe therapeutic challenge in CNSPA infections. Our results also affirm the limited use of C/T as an empirical agent in our setting, reserving the agent for culture-directed indications. Aside from polymyxin B and amikacin, which are often associated with toxicities, there are no safer and tolerable options for our multidrug-resistant CNSPA, prompting the urgent need to explore the use of other novel agents, such as cefiderocol or combination therapy, to fill the gaps in the armamentarium against CNSPA in our setting (21).

The high nonsusceptibility rates may be corroborated by our molecular findings. There is a high prevalence of well-established multidrug-resistant carbapenemase-producing P. aeruginosa high-risk clones (ST235, ST308, and ST179) among the C/T-nonsusceptible isolates. Additionally, diverse STs can also acquire ESBLs/carbapenemases, leading to reduced β-lactam susceptibilities. The role of constitutive AmpC variants leading to structural modifications and/or hyperproduction in mediating C/T resistance appeared to be minimal in our population. The *ampC* gene in P. aeruginosa is highly polymorphic, and mutations did not necessarily translate to changes in C/T phenotype.

A limitation of this study is that we did not measure the change in expression levels in AmpC. AmpC hyperproduction mediated by mutations in other unstudied genes may have been responsible for C/T resistance. Though they appear to be rare, we are not aware of the true proportion of AmpC hyperproducers in our population. However, more importantly, C/T resistance in strains not producing carbapenemases/acquired ESBLs predates the introduction of C/T into our clinical practice. This highlights that C/T resistance could result independently of C/T use, which could be due to the rampant use of other β-lactam antibiotics that are able to induce or derepress AmpC production (22). There is also a possibility that C/T resistance is independent of AmpC or *β-*lactamase activity. Mechanisms driving C/T resistance still need to be further explored.

Although C/T has been reported to be highly active against P. aeruginosa and retained susceptibility in CNSPA elsewhere in the world, susceptibility is not universal. The prevalence of C/T resistance is related to the molecular epidemiology of P. aeruginosa, which can vary temporally and geographically. In our setting, where prevalence of acquired *β*-lactamases is high, the utility of C/T is limited. Knowledge of the molecular epidemiology and genotypes is important in evaluating the place of therapy with novel agents.

## MATERIALS AND METHODS

### Collection of bacterial isolates.

Nonduplicate clinical CNSPA isolates, which exhibited nonsusceptibility to at least one carbapenem (doripenem, meropenem, imipenem), collected at the Singapore General Hospital (SGH) Pharmacy Research Laboratory between 2009 and 2020 from various culture sites (blood [53.5%], lower respiratory specimens [16.1%], skin and soft tissue [11.6%], urine [5.8%], and other sites, including bone, gastrointestinal tract, etc. [13.0%]), were studied. Isolates were randomly selected for testing from the laboratory’s repository, which comprised CNSPA isolates collected from an informal carbapenem-nonsusceptible Gram-negative pathogen surveillance study of hospital inpatients initiated in 2015. Isolates from prior to 2015 were collected via convenience sampling or were submitted to the laboratory for antibiotic combination testing.

These isolates were subjected to genus identification and confirmation as per the institution’s microbiology laboratory routine procedures, i.e., using Vitek GNI+ cards with the Vitek 2 instrument (bioMérieux, Hazelwood, MO) and matrix-assisted laser desorption ionization–time of flight mass spectrometry (MALDI-TOF MS) system (Bruker Daltonik, Germany), if necessary. All isolates were preserved in Microbank cryovials (Pro-Lab Diagnostics, Richmond Hill, ON, Canada) at −80°C and subcultured twice on Trypticase soy agar-5% sheep blood plates (BD, Sparks, MD) before experimental testing.

### Antibiotic susceptibilities.

Susceptibilities to meropenem, imipenem, doripenem, cefepime, piperacillin/tazobactam, levofloxacin, amikacin, and polymyxin B were determined using customized 96-well broth microdilution plates (TREK Diagnostics, East Grinstead, UK) in accordance with the manufacturer’s recommendations. Gradient MIC test strips were used to determine ceftazidime/avibactam (bioMérieux, Marcy l’Etoile, France) and ceftolozane/tazobactam (Liofilchem, Roseto degli Abruzzi, Italy) susceptibilities. Ceftazidime susceptibility was not routinely tested in this institution, as the agent was reserved primarily for the treatment of melioidosis. All MICs were interpreted according to the Clinical and Laboratory Standards Institute (CLSI) guidelines (23). P. aeruginosa ATCC 27853 was used as the quality control strain.

### DNA preparation and whole-genome sequencing.

Genomic DNAs were extracted and purified from overnight bacterial cultures with the DNeasy blood and tissue kit (Qiagen GmbH, Hilden, Germany) according to the manufacturer’s protocol. Paired-end whole-genome sequencing (WGS) was performed on the genomic DNAs using the MiSeq/HiSeq systems (Illumina Inc., CA), with a resultant coverage of at least 100-fold. Raw sequences were assessed for quality using FastQC (v0.11.3, Babraham Institute), followed by removal of adaptors and poor-quality bases using Trimmomatic (24, 25). Trimmed sequences were then assembled *de novo* using SPAdes software (26).

### Genotypic profiling.

Acquired resistance genes were identified using the SRST2 package (v0.2.0), which mapped raw short reads to the ARG-ANNOT database (27, 28). Selected chromosomal gene targets related to C/T susceptibility were analyzed by aligning assembled sequences to the PAO1 reference genome (GenBank accession no. AE004091.2), and variants were called with the pipeline Snippy (v4.6.0) (available at https://github.com/tseemann/snippy). The Protein Variation Effect Analyzer (PROVEAN) software tool was used to predict the impact of identified amino acid substitutions on protein biological function, i.e., whether the amino acid substitution was neutral or deleterious (http://provean.jcvi.org/index.php) (29). Sequence types (STs) were identified using the Basic Local Alignment Search Tool (BLAST) against the PubMLST database (https://pubmlst.org/paeruginosa/).

### Ethics statement.

This study is exempted from review by the Singhealth Centralised Institutional Review Board, as it is a retrospective study involving archival bacterial isolates, which does not fall under the Human Biomedical Research Act. No identifiable data were collected.

### Accession number(s).

Whole-genome sequences of the C/T-nonsusceptible CNSPA are available in the NCBI Sequence Read Archive (SRA) under BioProject accession number PRJNA656645.
